# Fluxomers: a new approach for ^13^C metabolic flux analysis

**DOI:** 10.1186/1752-0509-5-129

**Published:** 2011-08-16

**Authors:** Orr Srour, Jamey D Young, Yonina C Eldar

**Affiliations:** 1 Dept. of Electrical Engineering, Technion-Israel Institute of Technology, 32000 Haifa, Israel; 2 Department of Chemical and Biomolecular Engineering, Vanderbilt University, TN, USA

## Abstract

**Background:**

The ability to perform quantitative studies using isotope tracers and metabolic flux analysis (MFA) is critical for detecting pathway bottlenecks and elucidating network regulation in biological systems, especially those that have been engineered to alter their native metabolic capacities. Mathematically, MFA models are traditionally formulated using separate state variables for reaction fluxes and isotopomer abundances. Analysis of isotope labeling experiments using this set of variables results in a non-convex optimization problem that suffers from both implementation complexity and convergence problems.

**Results:**

This article addresses the mathematical and computational formulation of ^13^C MFA models using a new set of variables referred to as *fluxomers*. These composite variables combine both fluxes and isotopomer abundances, which results in a simply-posed formulation and an improved error model that is insensitive to isotopomer measurement normalization. A powerful fluxomer iterative algorithm (FIA) is developed and applied to solve the MFA optimization problem. For moderate-sized networks, the algorithm is shown to outperform the commonly used 13CFLUX cumomer-based algorithm and the more recently introduced OpenFLUX software that relies upon an elementary metabolite unit (EMU) network decomposition, both in terms of convergence time and output variability.

**Conclusions:**

Substantial improvements in convergence time and statistical quality of results can be achieved by applying fluxomer variables and the FIA algorithm to compute best-fit solutions to MFA models. We expect that the fluxomer formulation will provide a more suitable basis for future algorithms that analyze very large scale networks and design optimal isotope labeling experiments.

## Background

### Metabolic Pathway Analysis

Metabolism is the complete set of chemical reactions taking place in living cells. These chemical processes form the basis of all life, allowing cells to grow, reproduce, maintain their structure and respond to environmental changes. Metabolic reactions are divided into groups called metabolic pathways, which are typically constructed heuristically according to their connectivity and presumed function [1]. Each metabolic pathway is characterized by a set of chemical reactions that transform substrates into end products while generating intermediate byproducts. Due to its importance in medicine and biotechnology, metabolic pathway research has become a highly active field of investigation [2].

Initially, the structure of metabolic pathways was examined by identifying their intermediate compounds. Subsequently, the various biochemical reactions connecting these compounds were mapped. Due to the success of this research, the topological structure of many metabolic pathways is nowadays fully documented [3]. The next step in the progression of metabolic pathway research involves quantification of the rates of these various chemical reactions, also known as "fluxes". The values of these rates are affected by various environmental conditions and can change rapidly in response to perturbations. Nevertheless, if the environmental parameters are held fixed and stable, the network can attain a steady state in which the concentrations of all network metabolites are assumed constant over time. This, of course, implies that the rates of their input and output reactions must balance. The latter imposes a set of linear constraints on the metabolic fluxes, known as "stoichiometric balance equations" [4]. Unfortunately, since the number of unknown fluxes typically exceeds the number of independent stoichiometric balances, these constraints are insufficient to completely identify the metabolic network. In order to overcome this lack of information, additional constraints must be provided to the stoichiometric mathematical model to estimate the values of the network fluxes [5].

### ^13^C Isotope Labeling Experiments

Various experimental techniques have been developed to enable measurement of intracellular metabolic fluxes, either directly or indirectly. One of these approaches makes use of isotope labeling experiments. In this method, the metabolic system is administered a known amount of an isotopically labeled substrate (such as glucose labeled with ^13^C at specific atom positions). By measuring the resulting labeling patterns of intracellular metabolites after steady state has been achieved, additional flux information is obtained.

One major drawback of this experimental approach is the high complexity and computational intensity of the metabolic flux analysis (MFA) required to interpret these labeling measurements. In their series of articles, Wiechert et al. [6-9] constructed a systematic approach for performing this analysis. They show that measurements of the relative abundance of various isotope isomers, also known as "isotopomers", contain enough information to fully identify the metabolic fluxes of the network. Formulating the problem using isotopomer variables (or a transformed set of isotopomer variables referred to as "cumomers"), Wiechert et al. posed the flux estimation problem as a non-convex least-squares minimization, assuming random error is added to their isotopomer measurements. The resulting high-dimensional non-convex problem suffers from various drawbacks, such as slow convergence and notable probability of attaining local minima. Several optimization algorithms have been developed in order to address these drawbacks. Early approaches used iterative parameter-fitting algorithms [8], evolutionary algorithms [10] and simulated annealing [11]. Furthermore, several investigations have been conducted in order to assess the accuracy of these results [9,12,13]. Recently, a novel method to decompose the metabolic network into Elementary Metabolite Units (EMUs) was introduced [14] and implemented into the OpenFLUX software package [15]. This decomposition effectively reduces the size of the optimization problem by efficiently simulating only those isotopomers that contribute to the measurement residuals. Nevertheless, all of these algorithms suffer from several major drawbacks due to the standard isotopomer-flux variables used in formulating the optimization problem:

• *Presence of unstable local minima: *due to the non-convex nature of the objective function.

• *Complex mathematical representation and computational implementation*. This results in the need for ad-hoc algorithms and mathematical analysis, and long running times are required for reliable convergence.

The OpenFLUX implementation, for example, may require several dozens of convergence iterations with various initial values in order to achieve acceptable probability of obtaining the optimal set of fluxes in any one of its attempts. In addition, due to the chosen objective function, it is also commonly required to estimate *scaling factors *for each isotopomer measurement, because of the fact that the available experimental techniques are only capable of measuring isotopomer fractions up to a proportional scaling factor (see Mollney et al. [9] for further details).

### Our Contribution

This article introduces a new set of variables for simulating ^13^C isotope labeling experiments. The main idea underlying this reformulation is that, instead of treating fluxes and isotopomer variables separately, we identify a set of "isotopically labeled fluxes" as our state variables of interest. We refer to these variables as *fluxomers*. Fluxomers combine flux variables with isotopomer variables and consequently reduce the complexity and nonlinearity of the original isotopomer balance equations. In this article, we show that by reformulating the flux estimation problem in terms of fluxomer variables, it is possible to construct an algorithm that has the following key benefits:

• Provides efficient computation of *all *isotopomers in a metabolic pathway

• Is robust to measurement noise (i.e., suppresses the effects of measurement errors) and initial conditions

• Eliminates the need for measurement scaling factor estimation

• Poses the problem using simple mathematical expressions, allowing the use of generic optimization algorithms

The rest of the article is constructed as follows. The Results and Discussion section illustrates the advantage of our approach via simulation results comparing fluxomer variables to the commonly used cumomer approach and the more recently introduced EMU approach. The Methods section presents the detailed formulation of the fluxomers optimization problem and the fluxomers iterative algorithm (FIA) that provides a reliable and efficient method for solving it. All source code and executables for our algorithms are freely available at the author's website [16].

## Results and Discussion

We compared our FIA algorithm to the widely used MFA software 13CFLUX [17], which relies on the cumomer approach, and to the more recent Open-FLUX [15] software, which is based on the EMU [14] approach. In order to compare the methods, we conducted flux estimations for various well-studied metabolic pathways. Our first example is based upon the tutorial which Wiechert et al. provide with their 13CFLUX software: the *Embden-Meyerhof *and *Pentose Phosphate *metabolic pathways of *Escherichia coli *[17]. This example compares the running time and robustness of both algorithms in response to input noise. Our second example compares the results and performance of FIA to both an adhoc method and the OpenFLUX algorithm for the analysis of lysine production by *C. glutamicum*, as described by Becker et al. [18] and Quek et al. [15].

### FIA vs. 13CFLUX Comparison: Embden-Meyerhof and Pentose Phosphate Pathways

**Figure 1:**
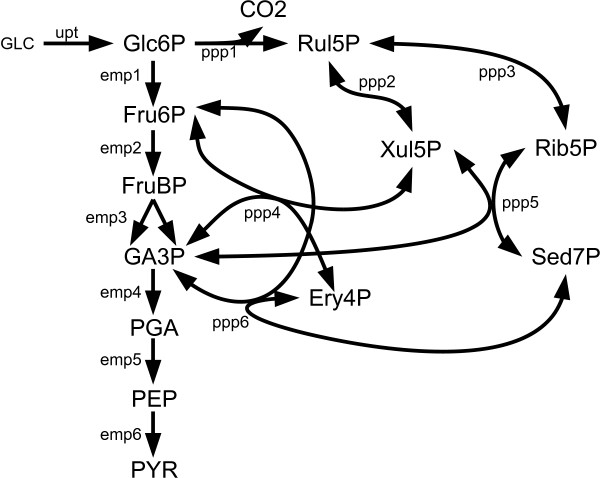
**E.Coli EMP and PPP Metabolic Pathways**. The *Embden-Meyerhof *and *Pentose Phosphate *metabolic pathways of *Escherichia coli*.

In this section we examine a network representing the *Embden-Meyerhof *and *Pentose Phosphate *pathways of *E. coli*, which is based upon the tutorial supplied by Wiechert et al. as part of their 13CFLUX software package. Since our FIA implementation natively supports 13CFLUX input files (i.e. "FTBL" files), the same input files can be used for both algorithms. (Note, however, that FIA does not require definition of free fluxes nor initial values, and thus these are simply ignored when imported). Figure 1 shows the simple network used along with the nomenclature used in previous publications. In addition to the network structure, the models are provided with flux and isotopic measurements as shown in Table 1.

**Table 1:** EMP & PPP simulation data

Label input data			
**Flux name**	**Cumomer Index**	**Value**	**STD**

GLC	#000000	0.445	-
	#100000	0.500	-
	#000001	0.011	-
	#000010	0.011	-
	#000100	0.011	-
	#001000	0.011	-
	#010000	0.011	-

Rul5P	#1xxxx	0.1979	0.002
	#x1xxx	0.0153	0.002
	#xx1xx	0.0284	0.002
	#xxx1x	0.0122	0.002
	#xxxx1	0.0976	0.002
Ery4P	#1xxx	0.0568	0.002
	#x1xx	0.0229	0.002
	#xx1x	0.0118	0.002
	#xxx1	0.0704	0.002
GA3P	#1xx	0.0330	0.002
	#x1x	0.0126	0.002
	#xx1	0.1207	0.002
PEP	#1xx	0.0330	0.002
	#x1x	0.0126	0.002
	#xx1	0.1207	0.002

Values are taken from the example input file included in the 13CFLUX demo. Substrate enrichment values are considered as constants.

First, we examined the output of the two algorithms for the traditional "noiseless" input file. In order to run the analysis, 13CFLUX requires the user to define a set of "free fluxes" along with their associated initial values [7]. Note that a bad choice of free fluxes or their associated values can result in poor algorithmic performance (both in computation time and accuracy). In fact, under various initial guesses the algorithm did not converge at all. As for FIA, none of the above is required. Since the network along with the given measurements are well defined, in the noiseless case the two algorithms returned similar values for unidirectional fluxes, as can be seen in Table 2. Some slight disagreements were observed for the bi-directional fluxes, which are more poorly identified.

**Table 2:** Comparison of FIA with 13CFLUX for the simple E.coli metabolic network

Flux name	FIA		13CFLUX	
		
	Est. flux	MSE	Est. flux	MSE
emp1	0.5100	0.0020	0.5099	0.0023
emp2	0.8500	0.0008	0.8500	0.0007
emp3	0.8500	0.0008	0.8500	0.0007
emp4	1.8700	0.0011	1.8700	0.0006
emp5	1.8700	0.0011	1.8700	0.0006
emp6	1.8700	0.0011	1.8700	0.0006
ppp1	0.5100	0.0019	0.5101	0.0023
ppp2	4.4234	0.5483	4.3281	0.9652
ppp2r	4.0834	0.5485	3.9880	0.9657
ppp3	4.4689	1.0365	2.7370	1.1057
ppp3r	4.2989	1.0368	2.5670	1.1057
ppp4r	4.0768	0.3643	4.1740	1.1608
ppp4	4.2468	0.3640	4.3440	1.1604
ppp5r	0.2538	0.1535	0.2680	0.0654
ppp5	0.4238	0.1531	0.4381	0.0655
ppp6r	0.2550	0.0175	0.2560	0.0194
ppp6	0.4250	0.0171	0.4260	0.0188
upt	1.0200	0.0004	1.0200	0.0001
coOut	0.5100	0.0019	0.5101	0.0023

Comparison of estimated fluxes and mean-square estimation error using "noiseless" data.

**Figure 2:**
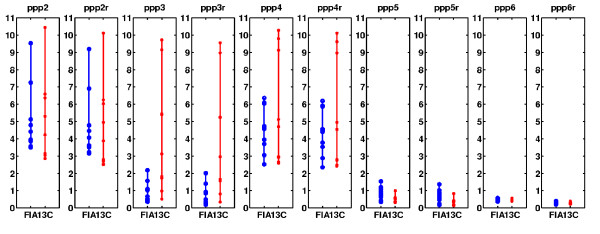
**Measured fluxes values**. Bidirectional fluxes calculated using FIA and 13CFLUX for noisy measurement set.

We next compared the algorithms' sensitivities to noise. In a series of 10 experiments, white Gaussian noise was added to all of the measured isotopomer values, and the outputs and computation times for both algorithms were recorded. As can be seen in Figure 2, unidirectional fluxes remain quite constant and hardly suffer from the added experimental error (for both algorithms). However, the bi-directional fluxes are affected by the added noise. 13CFLUX suffers from a higher variance spread of the estimated values than FIA (thus is more sensitive to the added measurement noise). Note that the difference arises not only due to the mathematical model used, but also due to the stability properties of the optimization method chosen.

We next examined the computational performance of the two methods. Table 3 shows the algorithm running time for convergence (in seconds). The average running time for 13CFLUX was 133 seconds, while for FIA this time was 7 seconds. The running time ratio (13CFLUX/FIA) for individual experiments varied between ×9 to ×75.

**Table 3:** Algorithm running time comparison for FIA vs. 13CFLUX

FIA	6.63	7.56	5.17	6.85	8.83	5.92	9.53	6.47	6.97	6.77
13CFLUX	59.14	56.93	76	121	65.7	451	81.7	173	177	69.65

Running time is shown in seconds.

### FIA vs. OpenFLUX Comparison: Lysine Production by C. glutamicum

In this section we examine the analysis of the central metabolism of two lysine-overproducing strains of *Corynebacterium glutamicum*: ATCC 13032 (lysC^fbr^) and its P_EFTU_fbp mutant. Both express feedback-resistant isoforms of the aspartokinase enzyme *lysC*, while the latter is additionally engineered to overexpress the glycolytic enzyme fructose-1,6-bisphosphatase. The example is based upon the measurements provided by Becker et al. [18], who implemented an ad-hoc program to estimate the values of various metabolic fluxes. In their more recent article introducing the OpenFLUX software package [15], Quek et al. chose to compare their results to those of Becker et al. Therefore, we will expand upon their comparison using our FIA implementation. The input file for FIA was constructed using the measurements and pathway structure given in [18] and [15]. As described in [15], the published mass isotopomer fractions were modified for mass interference from non-carbon backbone isotopes using the molecular formula of the amino acid fragments. FIA supports automatic generation of the naturally occurring isotopes correction matrix when the measured molecular formulas are supplied. This adjusts the measured fluxomers vector appearing in the objective function during the process of optimization. If necessary, it is possible not to use this feature but instead to directly supply the algorithm with the corrected measurement values.

When comparing the running times of FIA with OpenFLUX, the different algorithmic approaches of the two must be kept in mind. While OpenFLUX requires the user to supply it with sets of free fluxes, FIA requires no free fluxes nor initial values. Open-FLUX rapidly evaluates dozens of different optimization cycles with random initial values and seeks the best fitting result among them, while FIA uses only one single (longer) run. As such, the convergence probability of OpenFLUX depends on the number of attempts and random values generated during its operation, while the FIA results do not depend on any random value. Furthermore, in its analysis, EMU based algorithms evaluate only the fluxes necessary for measurement comparison, and thus their running time depends both on the metabolic network structure and the amount and location of the given measurements. FIA, on the other hand, can supply the entire set of metabolic fluxes at any given time, with no additional computation requirement (which depends mainly on the network structure).

#### Measured fluxes as constants

First, we ran the exact same simulation as Quek et al. performed in their article. They supply very accurate (mean error in the order of 0.15 mol%) values for the label measurements, and used the given measured fluxes as if they were noiseless measurements (thus as constants). We start by comparing the simulation time for this simple case. According to [15] and as validated by us using our computer, OpenFLUX required 50 iterations of about 16 seconds each in order to find a decent minimal point, hence about 800 seconds in total. While so, the FIA analysis took 60 seconds for initial analysis and matrices creation, and 300 further seconds for convergence, thus 360 seconds as a whole. Regarding the simulation results, as one can see in Table 4 and Table 5 the fluxes are very close to those calculated before, and the estimated fluxes FIA returned had the lowest residual value compared to the other methods.

**Table 4:** Relative mass isotopomer fractions comparison for wild-type and mutant C. glutamicum

		Wildtype							Mutant				
			
Fragment		Non-normalized	**Exp**.	Ad-hoc	OpenFLUX	FIA			**Exp**.	Ad-hoc	OpenFLUX	FIA	
										
						**const**.	**meas**.	ratios				**const**.	**meas**.
ALA 260	M0	206.3562	0.5085	0.509	0.509	0.5099	0.5099	0.5097	0.5230	0.525	0.525	0.5247	0.5247
	M1	102.8634	0.3529	0.354	0.354	0.3534	0.3534	0.3537	0.3410	0.342	0.342	0.3425	0.3425
	M2	4.8452	0.1058	0.106	0.106	0.1063	0.1063	0.1062	0.1030	0.104	0.104	0.1037	0.1037
VAL 288	M0	41.4005	0.3455	0.348	0.348	0.3459	0.3458	0.3457	0.3640	0.366	0.366	0.3661	0.3663
	M1	39.6134	0.3983	0.398	0.398	0.3986	0.3986	0.3987	0.3920	0.392	0.392	0.3921	0.3922
	M2	10.7340	0.1845	0.184	0.184	0.1846	0.1846	0.1847	0.1750	0.175	0.175	0.1750	0.1749
THR 404	M0	194.9082	0.3330	0.334	0.334	0.3343	0.3343	0.3340	0.3440	0.344	0.344	0.3439	0.3439
	M1	159.2226	0.3764	0.376	0.376	0.3757	0.3757	0.3759	0.3730	0.371	0.371	0.3715	0.3721
	M2	35.2094	0.1957	0.196	0.196	0.1956	0.1956	0.1957	0.1910	0.192	0.192	0.1920	0.1918
ASP 418	M0	159.9111	0.3343	0.333	0.333	0.3337	0.3337	0.3334	0.3450	0.343	0.343	0.3432	0.3433
	M1	128.3755	0.3732	0.375	0.375	0.3750	0.3750	0.3752	0.3700	0.370	0.371	0.3708	0.3714
	M2	28.7782	0.1955	0.196	0.196	0.1960	0.1959	0.1960	0.1920	0.193	0.192	0.1924	0.1922
GLU 432	M0	3.8009	0.2469	0.25	0.249	0.2474	0.2473	0.2469	0.2570	0.264	0.264	0.2634	0.2624
	M1	4.4232	0.3648	0.366	0.366	0.3661	0.3661	0.3660	0.3650	0.365	0.365	0.3656	0.3658
	M2	1.7429	0.2412	0.239	0.240	0.2406	0.2406	0.2409	0.2360	0.232	0.232	0.2322	0.2327
SER 390	M0	224.9043	0.4497	0.449	0.448	0.4487	0.4488	0.4490	0.4620	0.463	0.463	0.4635	0.4628
	M1	108.4056	0.3576	0.358	0.358	0.3578	0.3578	0.3580	0.3490	0.349	0.349	0.3491	0.3492
	M2	3.5199	0.1428	0.143	0.144	0.1437	0.1437	0.1434	0.1400	0.140	0.140	0.1399	0.1403
PHE 336	M0	250.7079	0.2712	0.274	0.274	0.2764	0.2764	0.2769	0.2870	0.289	0.289	0.2881	0.2874
	M1	303.6304	0.3816	0.381	0.381	0.3817	0.3817	0.3822	0.3800	0.381	0.381	0.3809	0.3806
	M2	129.5861	0.2282	0.228	0.228	0.2263	0.2264	0.2261	0.2200	0.220	0.220	0.2206	0.2210
GLY 246	M0	738.7580	0.7407	0.742	0.742	0.7417	0.7417	0.7421	0.7410	0.743	0.743	0.7426	0.7426
	M1	39.7395	0.1845	0.185	0.185	0.1852	0.1852	0.1849	0.1830	0.184	0.184	0.1844	0.1844
TYR 466	M0	36.7321	0.2344	0.236	0.236	0.2380	0.2380	0.2384	0.2460	0.249	0.249	0.2481	0.2475
	M1	43.7966	0.3530	0.356	0.356	0.3567	0.3567	0.3572	0.3510	0.358	0.357	0.3572	0.3569
	M2	18.6839	0.2423	0.245	0.245	0.2433	0.2433	0.2431	0.2340	0.238	0.238	0.2387	0.2390
TRE 361	M0	34.1048	0.0613	0.062	0.062	0.0612	0.0612	0.0608	0.0880	0.088	0.088	0.0884	0.0884
	M1	327.3441	0.6040	0.607	0.606	0.6051	0.6051	0.6057	0.5730	0.577	0.574	0.5743	0.5742
	M2	27.0318	0.2070	0.207	0.207	0.2084	0.2084	0.2084	0.2130	0.213	0.213	0.2128	0.2126

Sum of weighted residuals				761	684	654	650	718	1735	1461	1451	1308	

Experimental and calculated isotopomer MS fractions. The experimental data and ad-hoc simulation results are taken from Becker et al. [18]. The OpenFLUX results are taken from [15]. The simulated "non-normalized" data is generated by multiplying the given values after natural isotope correction by random factors. Several FIA estimations are provided: using the given fluxes as constants (under "const."), as measurements (under "meas."), and when using the simulated non-normalized data (under "ratios"). As can be seen, FIA agrees with previous results (even when the data is used without normalization). For the mutant case, better fits are achieved when allowing the supplied fluxes to change as well.

**Table 5:** Metabolic fluxes comparison for wild-type and mutant C. glutamicum

	Wildtype					Mutant			
		
	Becker	OpenFLUX	FIA			Becker	OpenFLUX	FIA	
						
			**const**.	**meas**.	ratios			**const**.	**meas**.
Glucose 6-phosphate isomerase	49.8	51.2	51.9	52.0	51.5	41.6	40.4	42.1	42.5
Glucose 6-phosphate dehydrogenase	46.8	45.0	44.7	44.7	45.1	56.2	57.5	55.7	55.1
Transaldolase	14	13.4	13.3	13.3	13.4	17.5	17.7	17.3	17.0
Transketolase 1	14	13.4	13.3	13.3	13.4	17.5	17.7	17.3	17.0
Transketolase 2	11.9	11.3	11.2	11.2	11.3	15.8	16.4	15.6	15.4
Glyceraldehyde 3-phosphate dehydrogenase	157.5	158.0	158.2	158.6	158.0	160.8	161.0	161.0	160.5
Pyruvate kinase	147.3	148.0	147.8	148.2	147.6	152.6	152.0	152.5	152.0
Pyruvate dehydrogenase	77.5	75.8	74.8	74.9	74.9	87.5	85.2	85.1	79.7
Pyruvate carboxylase - carboxykinase	34.4	35.8	35.9	36.1	35.8	31.5	32.4	32.5	34.9
Citrate synthase	52.5	50.8	49.6	49.7	49.9	67.7	65.4	65.3	58.9
Isocitrate dehydrogenase	52.5	50.8	49.6	49.7	49.9	67.7	65.4	65.3	58.9
Oxoglutarate dehydrogenase	41.2	39.4	38.2	38.3	38.5	59.9	57.6	57.5	50.7
Aspartokinase	11.2	11.2	11.2	11.4	11.2	14.2	14.2	14.2	15.9

Estimated metabolic fluxes values for the different approaches - the ad-hoc simulation results from Becker et al. [18], the OpenFLUX results [15], and the FIA results for its various simulated scenarios (measured fluxes used as constants, as measurements, and when using ratios of non-normalized data.)

#### Measured fluxes as measurements

We can also run the same optimization, but weight the given flux measurements by their variances. When running this optimization using OpenFLUX (again using 50 iterations), the amount of time was greatly increased, and ended in around 48 minutes. For FIA, on the other hand, the running time was the same as before, thus about 6 minutes. Comparing the results of the algorithms, OpenFLUX suffered from severe convergence problems. Most of its iterations ended without converging at all, while those that did converge yielded useless results, far from the measurements. FIA, on the other hand, succeeded in converging for all scenarios. For the wildtype lysine producing pathway, the results were very close to the ones before (since the fluxes and measurements were quite accurate). For the mutant example, which was less accurate, a reduction of the residual value was achieved by small changes to the measured fluxes. fluxes and residual values can be examined in Table 4 and Table 5.

#### Using non-normalized MS measurements

We now show that FIA can easily use incomplete or non-normalzied measurements by examining its performance in the example above. The supplied MS measurements were normalized to the *n *+1 backbone carbon atoms of the measured metabolites. Instead of using the supplied normalized data, we multiply each set of metabolite measurements by a random constant number. By doing so, we simulate the case in which only the first 3 (2 for GLY) MS peaks were measured, and had not been normalized. The original and supplied non-normalized measurement values can be found in Table 4. Note that the values were corrected by the molecular formulas of the measured fragments (again, can be automatically performed by FIA). In the absence of normalized data, FIA gave estimated fluxes very close to the previous cases, with very low residual values, as can be seen in Table 5. The running time of the algorithm was not affected by the change.

## Conclusions

The main contribution of this article is the introduction of *fluxomers*--a new set of state variables used to simulate ^13^C metabolic tracer experiments. The fluxomers approach allows the central optimization problem of MFA to be reformulated as a sequence of quadratic programs, which form the basis of the fluxomers iterative algorithm (FIA). Both fluxomers and FIA result in several important benefits compared to flux-isotopomer variables. Among these advantages are (i) a reduction in algorithm running time required for simulation of isotopomer distributions and metabolic flux estimation, (ii) reduced sensitivity to measurement noise and initial flux values and (iii) availability of complete isotopomer information for a given network (as opposed to the EMU approach, which only supplies partial information) without the need for user specification of free fluxes or initial flux values. Additionally, the error model used by the FIA algorithm has the advantage that it depends solely upon isotopomer *ratios *rather than complete isotopomer *fractions*, and therefore it eliminates the need to estimate a normalization factor for each measured isotopomer distribution. Our current results show significant improvements even with regards to simplistic tracer experiments (the running times have been improved by an order of ×3 to ×20 compared to the 13CFLUX algorithm, and about ×2 to ×8 compared to the OpenFLUX implementation). It is important to note that the total time required to obtain an MFA solution is controlled both by (i) the time of each iteration and (ii) the number of optimization iterations that are required to achieve a reliable solution. While a single OpenFLUX iteration is certainly faster than a single iteration of FIA, the FIA algorithm was expressly constructed to provide high reliability in achieving the optimal solution. Therefore, FIA was able to consistently find a better optimal solution in less total time in comparison to the other algorithms examined. Furthermore, extending the fluxomers formulation to other global optimization techniques is straightforward. We expect that reformulating more sophisticated MFA problems--for example, involving optimal experimental design or large-scale metabolic networks--in terms of fluxomer variables will lead to dramatic enhancements of algorithmic efficiency and robustness.

## Methods

**Figure 3:**
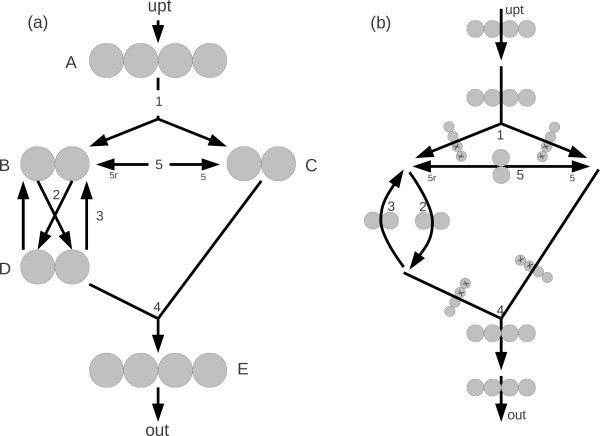
**Simple metabolic network**. (a) Standard network representation. Carbon atoms are drawn explicitly with arrows to indicate atom transitions. Unidirectional arrows represent unidirectional fluxes while bidirectional fluxes (such as flux 5) are represented by bidirectional arrows. (b) Fluxomers representation. Each arrow is a group of fluxomers. X's appear on the appropriate atom positions to indicate summation of divergent fluxomers.

In the following, we show how to construct and solve MFA problems using fluxomer variables. First we define and explain the basic properties of fluxomers. Then we show how to express MFA balance equations and measurements in terms of fluxomers. Finally, we formulate the MFA optimization problem and present the FIA algorithm for solving it. Throughout this section we use boldface uppercase letters **A **to denote matrices, lowercase boldface letters **x **to denote vectors, and lowercase letters *u *for scalars. We use the *<*○ *>*product **z **= **x**○**y **to represent the element-wise product vector, i.e. *z_i _*= *x_i_y_i_*. The model formulation will be illustrated using the simple metabolic network shown in Figure 3.

### Fluxomers overview

Traditional MFA approaches construct distinct variables for each flux and for each possible labeling state (isotopomer) associated with all metabolites in the network. Fluxomers, on the other hand, are a composite of these two and therefore allow the network state to be described using only one variable type.

**Definition 1 (Fluxomer) ***A fluxomer is the rate that a metabolic reaction transfers labeling from one or more specific substrate isotopomers into product isotopomers*.

Taking each fluxomer to be a transformation from one set of labeled atoms into others, we can write its labeling state as an array of binary elements representing the state of each atom it consumes (0 representing an unlabeled atom and 1 representing a labeled atom). Thus, *f_i_*(1001) is a fluxomer of reaction *i *consuming 4 atoms, with its first and last atoms labeled and two middle atoms unlabeled. When using *x *as an index for one (or more) of the atoms, we denote a sum of fluxomers where the indicated atom can be either labeled or unlabeled (e.g., *f_i_*(1*x*01) is the sum of *f_i_*(1001) and *f_i_*(1101)). See Figure 3b for a detailed example.

Traditional metabolic fluxes and isotopomer variables can be easily expressed using fluxomers. We start with metabolic fluxes, which are just a sum of their associated fluxomers. For the simple network in Figure 3b we have:

(1) $$\begin{array}{lll} f_{upt}=f_{1} & =\sum f_{1}\left(ijkl\right)=f_{1}\left(xxxx\right)\; & \text{(1)}\\ f_{2} & =\sum f_{2}\left(ij\right)=f_{2}\left(xx\right)\; & \text{(2)}\\ f_{3} & =\sum f_{3}\left(ij\right)=f_{3}\left(xx\right)\; & \text{(3)}\\ f_{out}=f_{4} & =\sum f_{4}\left(ijkl\right)=f_{4}\left(xxxx\right)\; & \text{(4)}\\ f_{5} & =\sum f_{5}\left(ijkl\right)=f_{5}\left(xxxx\right)\; & \text{(5)}\\ f_{5r} & =\sum f_{5r}\left(ijkl\right)=f_{5r}\left(xxxx\right).\; & \text{(6)}\\ & \; & \text{(7)} \end{array}$$

We can also express isotopomer abundances in terms of fluxomer variables for the same example. Because of the assumption that enzymes do not differentiate between the various isotopomers of a given metabolite, the isotopomers within each metabolite pool are distributed uniformly across the outgoing fluxes emanating from that pool. Therefore, the fractional abundance of a given isotopomer within a metabolite pool will determine the fractional contribution of its corresponding fluxomers to the fluxes leaving that pool:

(2) $$\begin{array}{c} A_{ijkl}\\ B_{ij}\\ C_{ij}\\ D_{ij}\\ E_{ijkl} \end{array}\begin{array}{c} =\\ =\\ =\\ =\\ = \end{array}\begin{array}{c} f_{1}\left(ijkl\right)∕f_{1}\\ f_{2}\left(ij\right)∕f_{2}=f_{5}\left(ij\right)∕f_{5}\\ f_{4}\left(xxij\right)∕f_{4}=f_{5r}\left(ij\right)∕f_{5r}\\ f_{4}\left(ijxx\right)∕f_{4}=f_{3}\left(ij\right)∕f_{3}\\ f_{out}\left(ijkl\right)∕f_{out}. \end{array}$$

### Fluxomer balance equations

We now examine the fluxomer balance equations that describe how fluxomers are propagated through the metabolic network. These balance equations represent the main mathematical device for calculating steady-state fluxomer values for a given network. For ease of notation, let us define the vector of metabolic fluxes in our system by $u\in R^{n}$ and the vector of fluxomers as $u\in R^{m}$. As shown above, the metabolic fluxes are calculated from a linear transformation of the fluxomers. Denoting this linear transformation matrix as **U**, we can write **u **= **Ux**. We now assume that we are given a certain **u **vector and wish to calculate the fluxomers in our system. We start by considering balances on "simple fluxomers", i.e. those that originate exclusively from a single metabolite pool. (An example of a simple fluxomer is *f*_5_(01) in Figure 3, which derives solely from pool B.) Under conditions of metabolic and isotopic steady state, the rate of 01-labeled molecules entering pool B must balance the rate that 01-labeled molecules leave that pool. Therefore, we can construct a balance on fluxomers around pool B as

(3) $$f_{5}\left(01\right)+f_{2}\left(01\right)=f_{1}\left(01xx\right)+f_{3}\left(01\right)+f_{5r}\left(01\right).$$

However, according to eq. 2 the left-hand side of this equation can be re-expressed as $B_{01}\left(f_{5}+f_{2}\right)=\frac{f_{5}\left(01\right)}{f_{5}}\left(f_{5}+f_{2}\right)$. Substituting this latter result into the flux balance equation and solving for the fluxomer *f*_5_(01) yields

(4) $$\begin{array}{lll} f_{5}\left(01\right) & =\frac{f_{5}}{f_{5}+f_{2}}\left(f_{1}\left(01xx\right)+f_{3}\left(01\right)+f_{5r}\left(01\right)\right)\; & \text{(1)}\\ & =g\left(u\right)\left(h^{T}x\right),\; & \text{(2)}\\ & \; & \text{(3)} \end{array}$$

where *g*(**u**) is a function of **u **alone, and **h **is a constant vector. Thus, for this simple case we can solve for the outgoing fluxomer *f*_5_(01) directly in terms of the fluxomers entering pool B and the total fluxes *f*_2 _and *f*_5 _leaving pool B.

We now turn to the more complex situation in which the output fluxomer originates from more than one metabolic pool. For example, consider fluxomer *f*_4_(0001) coming from pools *C *and *D*. Here, the fraction of 0001-labeling carried by flux *f*_4 _is proportional to the abundance of 01-labeling in C and 00-labeling in D:

(5) $$\begin{array}{lll} f_{4}\left(0001\right) & =f_{4}C_{01}D_{00}\; & \text{(1)}\\ & =f_{4}\left(\frac{f_{1}\left(xx01\right)+f_{5}\left(01\right)}{f_{4}}\right)\left(\frac{f_{2}\left(00\right)}{f_{3}+f_{4}}\right)\; & \text{(2)}\\ & =g\left(u\right)\left(h_{1}^{T}x\right)\left(h_{2}^{T}x\right).\; & \text{(3)}\\ & \; & \text{(4)} \end{array}$$

As before, the outgoing fluxomer *f*_4_(0001) can be expressed solely in terms of *g*--a pure function of **u **(always a rational function of outgoing fluxes)--and a product of linear projections of **x**.

Without loss of generality, we restrict ourselves to fluxes coming from at most 2 metabolic pools (referred to subsequently as the "left" and "right" pools). When the system reaches steady state, we have

(6) $$x=g\left(u\right)\circ \left(H_{1}x\right)\circ \left(H_{2}x\right),$$

where **g **is a function $R^{n}\to R^{m}$, and (**H_1_**, **H_2_**) are two *m *× *m *matrices. This equation allows for the output fluxomers emanating from a specific metabolite pool to be expressed in terms of the total flux vector **u **and the fluxomers entering the pool. This enables each outgoing fluxomer to be solved "locally" for the incoming fluxomers. Note that this local calculation does not involve any matrix inversions or other expensive computational procedures. If there are no recycle loops in the network so that all possible paths through the network are non-selfintersecting, this equation can be used to solve sequentially for all "downstream" fluxomers in terms of previously calculated "upstream" fluxomers. In the presence of recycle loops an iterative approach can be constructed to solve for the fluxomers while still avoiding repeated matrix inversions.

### Constructing the system matrices

The matrices **H_1_**, $H_{2}\in R^{mxm}$ are defined by

(7) $$\begin{array}{ll} {\left(H_{1}\right)}_{ij} & =1,\text{if}x_{j}\text{enterstheleft}(\text{for}H_{\text{2}},\text{right})\\ & \text{sourcemetabolicpoolinareactionforwhich}x_{i}\text{isaproduct}\\ {\left(H_{1}\right)}_{ij} & =0,\;\text{otherwise}. \end{array}$$

The function $g_{i}:R^{m}\to R^{n}$ is defined as

(8) $$g_{i}\left(u\right)=\frac{g_{1i}^{T}u}{\left(g_{2i}^{T}u\right)\left(g_{3i}^{T}u\right)},$$

with **g**_**1**i_, **g**_**2i**_, $g_{3i}\in R^{n}$ given by

(9) $$\begin{array}{ll} {\left(g_{1i}\right)}_{j} & =1,\;\;\text{if the fluxomer }x_{i}\text{ is part of the flux }f_{j},\;\\ {\left(g_{1i}\right)}_{j} & =0,\text{otherwise}\\ {\left(g_{2i}\right)}_{j} & =1,\;\;\text{if flux }f_{j}\text{ exits the left source pool}\\ & \text{in a reaction for which }x_{i}\text{ is a product},\\ {\left(g_{2i}\right)}_{j} & =0,\text{otherwise}\\ {\left(g_{3i}\right)}_{j} & =1,\;\;\text{if flux }f_{j}\text{ exits the right source pool}\\ & \text{in a reaction for which }x_{i}\text{ is a product},\\ {\left(g_{3i}\right)}_{j} & =0,\text{otherwise}\text{.} \end{array}$$

In matrix form,

(10) $$g\left(u\right)=\frac{G_{1}u}{\left(G_{2}u\right)\circ \left(G_{3}u\right)}.$$

### Isotopomer measurement formulation

In the following, we develop a systematic method for expressing measured isotopomer variables using fluxomer notation. The final result of the analysis shows that isotopomer measurements can be written simply as the norm of a linear transformation of fluxomers, thus *Err *~ ||**Ax**||^2^. First, we briefly summarize the available isotopomer measurements provided by Nuclear Magnetic Resonance (NMR) and Mass Spectrometry (MS) methods. We then discuss the mathematical modeling of these measurements using fluxomer variables.

#### Available isotopomer measurements

MFA experiments are typically carried out by (i) introducing a labeled substrate into a cell culture at metabolic steady state, (ii) allowing the system to reach an isotopic steady state, and (iii) measuring isotopomer abundances of metabolic intermediates and byproducts using either MS or NMR analysis. These two measurement techniques provide qualitatively different information about isotopic labeling.

• ^1^H NMR: Measures the fractional ^13^C enrichment of each proton-bound carbon atom, irrespective of the labeling of its neighboring carbon atoms. Both ^12^C and ^13^C atoms are distinguishable in the same spectrum, and therefore the peak areas corresponding to different carbon isotopes can be normalized directly.

• ^13^C NMR: Quantifies isotopomers based on the presence of multiplet peaks (e.g., doublets, triplets, doublet doublets, etc.) in the spectrum caused by two or more neighboring ^13^C atoms. Because ^12^C atoms are undetectable by ^13^C NMR, it is impossible to quantify the overall fraction of each isotopomer unless ^1^H NMR spectra are simultaneously obtained. Instead, only the relative ratio of different isotopomers can be assessed by ^13^C NMR.

• MS: This technique is usually preceded by some form of chromatographic separation (GC or LC) to resolve mixtures into their individual components. These components are then ionized and fragmented in the MS ion source. The ionized particles are separated according to their masses by an electromagnetic filter, and a detector measures the relative abundance of each mass isotopomer. These abundances can be normalized to a fractional scale if all MS peaks corresponding to a particular ion are simultaneously measured.

Previous studies based on flux-isotopomer variables have modeled the measurement error as Gaussian noise added to the fractional isotopomer enrichments. Therefore, if $\hat{y}$ is the vector of measured isotopomer fractions, this model states that $\hat{y}=y+e$, where **e **is the Gaussian error term. However, a more accurate error model would add the measurement noise directly to the physically measured values. The motivation for the traditionally chosen error model is its relative simplicity when expressed using flux-isotopomer variables. Furthermore, since some isotopomers of a specific metabolite may be unmeasurable, the isotopomer fractions cannot be experimentally determined in many cases. This implies the need for an alternative error model that avoids these shortcomings.

#### Measurement Error Model

We denote the measured isotopomer abundances by a vector $\hat{m}$. For NMR analysis, the elements of $\hat{m}$ are proportional to the areas under the different spectral peaks. For MS, they are proportional to the integrated ion counts associated with each mass isotopomer. Since $\hat{m}$ is the measured quantity, the correct error model is an addition of Gaussian noise so that $\hat{m}=m+e$, where **m **is the "true" measurement value. The measured isotopomer fractions $\hat{y}$ are then expressed as

(11) $${ŷ}_{j}=\frac{{\hat{m}}_{j}}{\sum_{i}{\hat{m}}_{i}}=\frac{m_{j}+e_{j}}{\sum_{i}\left(m_{i}+e_{i}\right)}.$$

Let *ε_j _*represent the residual between the modelpredicted and experimentally measured abundance of a single isotopomer. After multiplying eq. 11 by $\sum_{i}\left(m_{i}+e_{i}\right)$ and rearranging, the residual expression becomes

(12) $$\varepsilon_{j}=m_{j}-{ŷ}_{j}\left(\underset{i}{\sum }m_{i}\right)=e_{j}-{ŷ}_{j}\underset{i}{\sum }e_{i},$$

where *ε_j _*is a sum of Gaussian variables. Noting that each measurement *m_j _*is simply proportional to a linear combination of fluxomers, the residual expression eq. 12 takes the form

(13) $$\varepsilon =\left[diag\left(\hat{y}\right)T-V\right]x,$$

where **T **and **V **are transformation matrices needed to convert fluxomers to isotopomer measurements and the **diag **operator converts its vector argument into a diagonal matrix. The resulting expression is both a simple sum of Gaussian vectors and affine in **x**.

The advantage of this objective function is that it only depends upon the relative isotopomer intensities in the vector $\hat{y}$ but does not depend upon how these intensities have been normalized (as long as the transformation matrix **T **is constructed accordingly). This eliminates the need to estimate optimal normalization factors that are required by previous algorithms in order to convert experimental measurements into isotopomer fractions. This is true for both MS and NMR measurements, either when conducted alone or used together in the same experiment.

### The MFA optimization problem using fluxomers

Now that we have defined both the isotopomer measurements and the feasible solution set, we can formulate the least-squares MFA optimization problem in terms of fluxomer variables. Our objective is to find the flux vector **u **that minimizes the measurement error. In addition to the fluxomer balances, usually upper bounds **u_ub _**are provided for all fluxes. As has been proven by Wiechert et al. [6-9], once the inputs to the system and **u **are set, the solution (**x**, **u**) is unique. In other words, the steady-state fluxomer balance equation, eq. 6, is actually an implicit definition of **x**(**u**). With this in mind, the MFA optimization problem can be simply defined as

(14) $$\underset{u\in Q}{\min}€€Ax(u)-b€{€}^{2}$$

with

$$\begin{array}{cc} Q= & \left\{\begin{array}{cc} u: & \begin{array}{c} Su=0\\ 0\leq u\leq u_{ub} \end{array} \end{array}\right\}, \end{array}$$

where **A **selects the measured elements of the fluxomers vector **x**(**u**), **b **contains their associated values, and **S **is the stoichiometric matrix of the reaction network. Note that **b **may contain non-zero elements only when associated with measurements of absolute flux values. For isotopomer measurements, the associated elements of **b **are zero.

Eq. 14 can be solved using various non-convex global optimizing techniques. These optimizers typically require the user to provide subroutines for computing the value of the objective function and its first derivatives at various points along their convergence path. Furthermore, evaluation of the function **x**(**u**) and its derivatives are the main (practically only) time-consuming procedures when solving the optimization problem in eq. 14. The mathematical formulation of eq. 14 is similar to the optimization problem resulting when using the labels and fluxes variables, with one exception - the implicit formula for **x**(**u**). As shown above, using fluxomers we are able to formulate the propagation equation (and thus solving **x**(**u**)) as a multiplication of homogeneous functions of fluxes, and second order functions of fluxomers. Using labels and fluxes, formulating the same equation results in a sum of functions of the same structure, and the homogeneous separation property vanishes. The following sections exploit this unique property of the fluxomers propagation equation in order to achieve great reduction in the system computational complexity, leading to the FIA algorithm.

### Fluxomers Iterative Algorithm (FIA)

This section deals with the evaluation of **x**(**u**) along with its gradient using the fluxomer formulation. First, we show that **x**(**u**) can be calculated iteratively while avoiding repeated matrix inversions. Then, we demonstrate how the number of iterations can be reduced using a Newton-type gradient-based algorithm. Finally, we explain how it is possible to greatly increase the sparsity of the system using a simple linear transformation of variables, which further reduces the number of iterations needed for convergence.

#### Solving the fluxomer balance equations

A simple approach for computing **x **given **u **is to imitate nature. Once a metabolic network reaches steady state (namely, when **u **is constant), changing its input labeling does not affect its flux values **u**, but only influences the labeling of its intermediate metabolite pools. The metabolite labeling patterns become gradually mixed and propagated throughout the network until isotopic equilibrium is reached. Accordingly, a simple approach for solving eq. 6 is by using its iterative representation (which is similar to the process taking place in nature):

$$x_{t+1}=g\left(u\right)\circ \left(H_{1}x_{t}\right)\circ \left(H_{2}x_{t}\right),$$

where **x_t _**is the fluxomer vector at iteration *t *and **x**_**t**+**1 **_is the fluxomer vector at iteration *t *+ 1. In order to simulate the steady-state labeling, we initialize the system with the vector **x_0 _**in which only the input fluxomers are labeled and all others are unlabeled. By recursively substituting **x **back into the equation, steady state is eventually reached and the final value of **x **is obtained. (This equation represents a non-linear time-invariant Markov chain.) For the *Embden-Meyerhof *and *Pentose Phosphate Pathway *example in the Results and Discussion section, it takes a few hundred iterations to achieve complete stability of the solution (maximal fluxomer value change on the order of 1e-17). Algorithm convergence for a given input vector is retrieved exactly as in the real biological system, and thus a unique solution always exists (for realistic metabolic networks).

We now show it is possible to reach pathway convergence in much fewer iterations. First, we write eq. 6 as

(15) $$F\left(x,u\right)=g\left(u\right)\circ \left(H_{1}x\right)\circ \left(H_{2}x\right)-x.$$

Now, in order to find the values of (**x**, **u**) one needs to solve **F**(**x**, **u**) = 0 while holding **u **constant. We choose to use one of the classic and powerful algorithms for finding roots of an equation, the well known Newton-Raphson [19-21] method. Roughly speaking, the change of the **x **vector at each iteration is calculated by

$$x_{t+1}=x_{t}-(F_{x}^{\prime }{\left(x,u\right)}^{-1}F(x,u),$$

with $F_{x}^{\prime }\left(x,u\right)=\frac{\partial F\left(x,u\right)}{\partial x}$. The main concern now is the evaluation of the expression ${\left(F_{x}^{\prime }(x,u)\right)}^{-1}F(x,u)$. Here, it turns out that due to the decomposable nature of **F**(**x**, **u**), the derivative $F_{x}^{\prime }$ at a point (**x**, **u**) is the simple matrix

(16) $$\begin{array}{lll} F_{x}^{\prime }\left(x,u\right) & =\left(g\left(u\right)\circ \left(H_{1}x\right)\right)H_{2}\; & \text{(1)}\\ & +\left(g\left(u\right)\circ \left(H_{2}x\right)\right)H_{1}-I.\; & \text{(2)}\\ & \; & \text{(3)} \end{array}$$

Therefore, finding $\text{r}={\left(F_{x}^{\prime }(x,u)\right)}^{-1}F(x,u)$ is equivalent to solving the linear system of equations

(17) $$(F_{x}^{\prime }(x,u))\text{r}=F(x,u).$$

In order to determine the root of the propagation equation, FIA starts with an iteration or two using Newton's correction and then continues with the simple "natural" approach. Applying this method to the *Embden-Meyerhof *and *Pentose Phosphate Pathway *example in the Results and Discussion section, only a few dozen iterations are now needed. In the next section we show how to reduce both the number of variables and the number of iterations required for convergence by another order of magnitude, without affecting system convergence stability.

#### Reducing system complexity

The following section introduces a mathematical approach for reducing the number of nonzero elements in our system. Variable reduction techniques such as the recently developed Elementary Metabolite Unit (EMU) network decomposition [14] were developed for application to systems that are modeled using flux-isotopomer variables. Fluxomers and the FIA algorithm, as opposed to prior approaches, allow us to effectively reduce the number of system variables using a simple linear transformation on **x**. Our main goal here is to find a transformation for the fluxomer vector **x**, **y **= **Kx **that:

• Reduces the number of its nonzero elements.

• Reduces the computational complexity of solving eq. 16.

• Eases the evaluation of eq. 15.

From eq. 16 we see that the greatest expense is due to inversion of a sum of two linear transformations (**H_1 _**and **H_2_**) of **x**. From the iterative propagation equation, eq. 15, we see that **x **is iteratively calculated by computing the product of the same two matrices. Had it been possible to find a sparse, close-to-diagonal representation for both **H_1 _**and **H_2 _**by simply multiplying them by the matrix from the right, both problems would be solved.

In order to acomplish the above, we examine the properties of the concatenation of these two matrices which we denote by **H**. Next we find the LU factorization of **H**,

(18) $$\left(\begin{array}{c} H_{1}\\ H_{2} \end{array}\right)=H=L_{H}U_{H}=\left(\begin{array}{c} L_{H1}\\ L_{H2} \end{array}\right)U_{H},$$

with **L_H _**lower triangular and **U_H _**upper triangular matrices. The matrix **L_H1 _**contains the first *m *rows of **L_H _**and **L_H2 _**contains the last *m *rows of **L_H_**. Our new set of variables now becomes **y **= **U_H_x**, and the new propagation equation is

(19) $$U_{H}\left[g\left(u\right)\circ \left(L_{H1}y\right)\circ \left(L_{H2}y\right)\right]-y=0.$$

**Figure 4:**
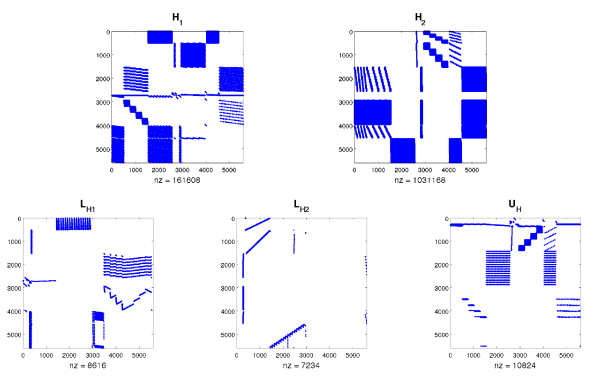
**System matrices complexity reduction**. **H_1_**, **H_2_**, **L_H1_**, **L_H2 _**and **U_H _**for the simple *E. coli *example. A substantial reduction in nonzero elements between the *H *and *L *matrices can clearly be seen.

When expressed in terms of the variable **y**, our system becomes much more sparse. This is illustrated in Figure 4 which shows **H_1_**, **H_2_**, **L_H1_**, **L_H2 _**and **U_H _**for the *Embden-Meyerhof *and *Pentose Phosphate Pathway *example. The transformation has two essential benefits:

1. Reduced computational complexity -- note that the derivative $F_{x}^{\prime }$ depends upon the matrices **H_1 _**and **H_2 _**which have already been factored, and thus solving Newton's step is straightforward.

2. Fewer iterations needed for convergence.

As a matter of fact, this transformation reduced the number of iterations needed for convergence of the simple *E. coli *example to a total of 5.

*Finding *$\frac{\partial x}{\partial u}$

As discussed above, our optimization problem seeks the minimum of ||**Ax**(**u**) - **b**||^2^. In order to converge rapidly, the gradient of the objective function must be computed at each iteration of the algorithm. The key step for computing it is the evaluation of $\frac{\partial x}{\partial u}$ (the derivative of the fluxomers as a function of the metabolic fluxes). Since we have an implicit function **F**(**x**, **u**) along with a valid solution for **F**(**x**, **u**) = 0, the implicit function theorem [22,23] can be used to compute $\frac{\partial x}{\partial u}$. Because **F**(**x**, **u**) is continuously differentiable around its root, we can write

(20) $$\frac{\partial x}{\partial u}=-{\left(\frac{\partial F\left(x,u\right)}{\partial x}\right)}^{-1}\frac{\partial F\left(x,u\right)}{\partial u}.$$

In the previous section we showed that $\frac{\partial F\left(x,u\right)}{\partial x}$ can be directly expressed in terms of the system matrices and the vectors **x **and **u**. The same procedure can be carried out in order to determine $\frac{\partial F\left(x,u\right)}{\partial u}$

(21) $$\frac{\partial F(x,u)}{\partial u}=diag((H_{1}x)\circ (H_{2}x))[\frac{G_{1}}{\left(G_{2}u)\circ (G_{3}u\right)}+\frac{((G_{1}u)\circ (G_{2}u))G_{3}+((G_{1}u)\circ (G_{3}u))G_{2}}{{\left(\left(G_{2}u)\circ (G_{3}u\right)\right)}^{2}}].$$

Keeping in mind that **F_x_**(**x**, **u**) is in its reduced form due to our variable transformation, solving the equation $\left(\frac{\partial F\left(x,u\right)}{\partial x}\right)\left(\frac{\partial x}{\partial u}\right)=-\frac{\partial F\left(x,u\right)}{\partial u}$ can be accomplished efficiently.

#### The initial point

The generation of the initial point for the FIA algorithm is very similar to the standard method used by many iterior point algorithms for finding a valid initial point over a convex linear set. We added a fluxes-measurement regularization factor *λ *in order to generate an initial point closer to the final estimation (and thus speed up the convergence process). The initial point is generated by solving the following simple convex optimization problem:

$$\underset{u,s\in \varepsilon }{\min}(-s+\lambda ∥Au-\hat{u}{∥}^{2}),$$

with

$$=\left\{\begin{array}{cc} u: & \begin{array}{c} Su=0\\ \left[I,-I\right]u+\left[I,I\right]{\left[0,u_{b}\right]}^{T}\geq s \end{array} \end{array}\right\}$$

with **A **a matrix that selects the measurable elements of **u**, $\hat{u}$ the meaured elements of **u **(if they exist), **0 **a vector of zeros, and **u_b _**a vector of the flux upper bounds. The regularization factor *λ *starts with some large value, and if necessary is reduced until the optimal value of *s *is greater than 0. Note that when *λ *→ 0 the problem reduces to finding a feasibile solution of **u**, and thus always has a solution (for well-structured networks).

#### The algorithm

Summarizing the above discussion leads to the following algorithm for efficient solution of the MFA optimization problem using fluxomers:

i. Matrix preparation (run once per network):

0. Calculate $\left(\begin{array}{c} H_{1}\\ H_{2} \end{array}\right)=\left(\begin{array}{c} L_{H1}\\ L_{H2} \end{array}\right)U_{H}$ using LU (PQ) factorization.

ii. Call the optimizer in order to solve

$$\underset{u\in Q}{\min}∥Ax(u)-b{∥}^{2},\;Q=\left\{\begin{array}{cc} u: & \begin{array}{c} Su=0\\ 0\leq u\leq u_{ub} \end{array} \end{array}\right\}$$

**When requested by the optimizer, return x**(**u**) **and its first derivative:**

1. Set **y_0 _**= **y_init_**.

2. Set *i *= 1.

3. Calculate

**y_i _**= **U_H _**[**g**(**u**) ○ (**L_H1_y_i - 1_**) ○ (**L_H2_y_i - 1_**)].

4. If ||*y*_*i *_- *y*_*i *- 1_||^2 ^>*ε*_*N*_

(a) Solve $(F_{x}^{\prime }(x,u))\text{r}=F(x,u).$

(b) Set **y_i _**= **y_i _**- **r **according to Newton's method.

5. If ||*y*_*i *_- *y*_*i *- 1_||^2 ^*> ε_e _*go to 3.

6. Calculate **x **= [**g**(**u**) ○ (**L_H1_y_i_**) ○ (**L_H2_y_i_**)].

7. Solve $\left(\frac{\partial F\left(x,u\right)}{\partial x}\right)\left(\frac{\partial x}{\partial u}\right)=-\frac{\partial F\left(x,u\right)}{\partial u}$.

The supplied software uses a variant of the "sequential least-squares" algorithm [24,25] for solving the non-convex optimization problem in eq. 14. This essentially breaks the problem into a sequence of convex optimization problems for which the solution can be readily computed. Note that other algorithms can be easily used with the same procedures described above.

## Authors' contributions

OS developed and implemented the theory, algorithm, simulations and software. YCE and JDY initiated and supervisied the study, and contributed to writing the manuscript. All authors reviewed, edited and approved the final manuscript.
